# Wnt16 Elicits a Protective Effect Against Fractures and Supports Bone Repair in Zebrafish

**DOI:** 10.1002/jbm4.10461

**Published:** 2021-02-02

**Authors:** Lucy M McGowan, Erika Kague, Alistair Vorster, Elis Newham, Stephen Cross, Chrissy L Hammond

**Affiliations:** ^1^ School of Physiology, Pharmacology and Neuroscience University of Bristol Bristol UK; ^2^ Wolfson Bioimaging Facility University of Bristol Bristol UK

**Keywords:** FRACTURE HEALING, GENETIC ANIMAL MODELS, OSTEOBLASTS, OSTEOPOROSIS, WNT

## Abstract

Bone homeostasis is a dynamic, multicellular process that is required throughout life to maintain bone integrity, prevent fracture, and respond to skeletal damage. *WNT16* has been linked to bone fragility and osteoporosis in human genome wide‐association studies, as well as the functional hematopoiesis of leukocytes *in vivo*. However, the mechanisms by which *WNT16* promotes bone health and repair are not fully understood. In this study, CRISPR‐Cas9 was used to generate mutant zebrafish lacking Wnt16 (*wnt16* ^*−/−*^) to study its effect on bone dynamically. The *wnt16* mutants displayed variable tissue mineral density (TMD) and were susceptible to spontaneous fractures and the accumulation of bone calluses at an early age. Fractures were induced in the lepidotrichia of the caudal fins of *wnt16* ^*−/−*^ and WT zebrafish; this model was used to probe the mechanisms by which Wnt16 regulates skeletal and immune cell dynamics *in vivo*. In WT fins, *wnt16* expression increased significantly during the early stages for bone repair. Mineralization of bone during fracture repair was significantly delayed in *wnt16* mutants compared with WT zebrafish. Surprisingly, there was no evidence that the recruitment of innate immune cells to fractures or soft callus formation was altered in *wnt16* mutants. However, osteoblast recruitment was significantly delayed in *wnt16* mutants postfracture, coinciding with precocious activation of the canonical Wnt signaling pathway. *In situ* hybridization suggests that canonical Wnt‐responsive cells within fractures are osteoblast progenitors, and that osteoblast differentiation during bone repair is coordinated by the dynamic expression of *runx2a* and *wnt16*. This study highlights zebrafish as an emerging model for functionally validating osteoporosis–associated genes and investigating fracture repair dynamically *in vivo*. Using this model, it was found that Wnt16 protects against fracture and supports bone repair, likely by modulating canonical Wnt activity via *runx2a* to facilitate osteoblast differentiation and bone matrix deposition. © 2021 The Authors. *JBMR Plus* published by Wiley Periodicals LLC. on behalf of American Society for Bone and Mineral Research.

## Introduction

The maintenance of skeletal health is central to many essential processes in the body. In addition to facilitating movement and protecting vital organs, bones regulate mineral reserves, hematopoiesis, and influence systemic hormone levels.^(^ ^1^ ^)^ Skeletal homeostasis is maintained by numerous cell types such as chondrocytes, osteoblasts, osteocytes, osteoclasts, and innate immune cells.^(^ ^2^, ^3^ ^)^ These cell types act in concert to maintain an optimal balance between bone deposition and bone resorption under steady‐state conditions and respond to acute skeletal damage such as fracture.^(^ ^4^ ^)^ Osteoporosis occurs when bone deposition is reduced in relation to bone resorption, resulting in low BMD and loss of bone integrity.^(^ ^3^ ^)^ Poor bone quality and low BMD is a strong predictor of fracture risk.^(^ ^5^ ^)^ Currently, an estimated 3.5 million people in the United Kingdom suffer from osteoporosis, resulting in over half a million fractures per year.^(^ ^6^ ^)^ Fragility fractures cause extensive morbidity and pose a high socioeconomic burden. As the aging population increases, the treatment costs associated with osteoporotic bone fractures are set to rise by 30% in the next decade. Hence, there is an urgent unmet demand to understand the underlying causes of osteoporosis, identify novel targets for therapeutic intervention, and promote optimal bone repair postfracture.

Wnt signaling pathways are highly conserved, central regulators of skeletal development and homeostasis, which act on bone throughout the lifetime of vertebrate organisms.^(^ ^7^ ^)^ Canonical Wnt pathway activation leads to the stabilization of β‐catenin and activation of transcription factors, whereas the calcium‐dependent and planar cell polarity noncanonical Wnt signaling pathways regulate intracellular calcium levels and Jun N‐terminal kinase (JNK) activity, respectively.^(^ ^8^ ^)^ Wnt ligands are a family of secreted glycoproteins that influence cell stemness, proliferation, differentiation, and migration via Wnt signaling pathways. WNT16 is one such ligand that can influence the activity of canonical and noncanonical Wnt pathways.^(^ ^9^, ^10^ ^)^ Recently, WNT16 has emerged as a regulator of cortical bone thickness and BMD, with mutations in *WNT16* linked to osteoporosis susceptibility in human genome wide‐association studies (GWASs).^(^ ^11^, ^12^ ^)^ Furthermore, a meta‐analysis of GWASs in women aged 20 to 45 years also associated *WNT16* with lumbar‐spine BMD, indicating that *WNT16* may influence BMD throughout life, not only in postmenopausal populations.^(^ ^13^ ^)^

Current experimental evidence highlights WNT16 as a potential regulator of bone homeostasis and repair, as well as immune cell development. Knockout of *Wnt16* in mice has been shown to lead to decreased cortical bone thickness and up to a 61% decrease in femur and tibia bone strength compared with WT littermates in three‐point bending tests.^(^ ^14^ ^)^ Although the loss of *Wnt16* in mice decreases bone strength, overexpression of *Wnt16* in osteoblasts (under the *Col1a1* promoter) leads to increased bone formation.^(^ ^15^, ^16^ ^)^ However, one study showed that *Wnt16* overexpression in osteoblasts could not counter glucocorticoid‐induced osteoporosis and bone loss, suggesting that other factors play a role.^(^ ^16^ ^)^ One possible explanation could include interactions with the immune system. Glucocorticoid treatment in zebrafish has been shown to suppress the innate immune system and osteoblast activity, leading to decreased bone synthesis.^(^ ^17^ ^)^ It has also been shown that morpholino–mediated knockdown of *wnt16* in zebrafish embryos results in impaired hematopoiesis and loss of thymic T lymphocytes at 4 days postfertilization (dpf).^(^ ^18^ ^)^ Embryonic knockdown experiments have shown that somatic *wnt16* expression is required for the upregulation of notch ligands and subsequent expression of the hematopoietic stem cell (HSC) marker *cd41*, which is needed for proper immune cell differentiation.^(^ ^18^ ^)^ Despite its proposed role in early HSC development, the relationship between Wnt16 and the immune system has not been explored further in adult tissues or in stable mutant lines. Moreover, there is increasing interest in the interplay between immune cells and bone; osteoclasts and macrophages are derived from a common myeloid progenitor cell population, and it is thought that macrophages can differentiate directly into osteoclasts in response to environmental molecular stimuli.^(^ ^19^ ^)^ The rapid but tightly regulated recruitment of innate immune cells is also required for optimal bone repair postfracture.^(^ ^20^, ^21^ ^)^ WNT16 has been linked to bone maintenance, fracture susceptibility, and leukocyte differentiation. However, functional studies to elucidate the role of WNT16 in these dynamic processes are still required.

Zebrafish (*Danio rerio*) serve as excellent models for studying the musculoskeletal system and innate immunity. Approximately 85% of human disease‐related genes have an ortholog in zebrafish.^(^ ^22^ ^)^ As a result, many of the developmental processes, cell types, and immune cell populations contributing to bone maintenance in humans are strongly conserved.^(^ ^23^ ^)^ Crucially, transparent zebrafish fin tissue provides optical clarity for high‐quality, dynamic live imaging of adult bone tissue and injury repair *in vivo*. Recently, the crushing of zebrafish caudal fin ray bones (lepidotrichia) was established as a model for studying fracture repair *in vivo*.^(^ ^24^ ^)^ Therefore, we used CRISPR/Cas9 technology to generate a stable *wnt16* ^*−/−*^ mutant line of zebrafish to investigate how loss of functional *wnt16* would affect bone maintenance, fracture repair, and innate leukocyte function. We show that the lack of Wnt16 in zebrafish leads to variable TMD in the fins and increased frequency of spontaneous fractures of caudal lepidotrichia in early adulthood and that *wnt16* is significantly upregulated in the bone of WT zebrafish postfracture. We employed an induced fracture model to further characterize key immunological and osteological events underpinning bone repair in zebrafish. We show that *wnt16* ^*−/−*^ zebrafish repair bone more slowly compared with WT zebrafish. Surprisingly, the recruitment of innate immune cells (neutrophils and macrophages) was unaffected by loss of Wnt16 postfracture. We found no measurable difference in overall osteoclast activity (tartrate‐resistant acid phosphatase [TRAP] staining) but observed more distinct, concentrated areas of TRAP^+^ punctae in *wnt16* mutant fractures. Impaired fracture healing in *wnt16* ^*−/−*^ zebrafish coincided with higher levels of canonical Wnt activation and delayed osteoblast recruitment, but no difference in soft callus formation was observed. We show that canonical Wnt‐responsive cells in the fracture are likely osteoblast progenitors. Taken together, our data suggest that Wnt16 promotes optimal bone repair postfracture by regulating osteoblast differentiation and bone matrix synthesis via the regulation of canonical Wnt activity and *runx2a*. This highlights the modulation of the canonical Wnt pathway and WNT16 as potential osteo‐anabolic candidates for further exploration in osteoporosis therapy development. Our data also further promote zebrafish as an emerging model for the dynamic study of fracture repair *in vivo* and for the rapid validation of human osteoporosis–associated genes.

## Materials and Methods

### Animal husbandry and transgenic zebrafish lines

All zebrafish were maintained at the University of Bristol's Animal Scientific Unit as previously described.^(^ ^25^ ^)^ Experiments were approved by the local ethical committee (the Animal Welfare and Ethical Review Committee for the University of Bristol, UK) and performed under a UK Home Office project license. The transgenics used have been previously described (Table 1).

**Table 1:** Transgenic Lines as Listed on zfin.org and Abbreviations Used in Text

Line name and reference	Abbreviation	Description
Tg*(7xTCF‐Xla:Siam:nlsGFP)* ^(^ ^26^ ^)^	Wnt:GFP	Canonical Wnt activity
*osx‐nls:eGFP* ^(^ ^27^ ^)^	*osx*:GFP	Osteoblasts
*Tg(col2a1aBAC:mCherry)* ^(^ ^28^ ^)^	*col2a1*:mCherry	Chondrocytes
*Tg(mpeg1:mCherry)* ^(^ ^29^ ^)^	*mpeg1*:mCherry	Macrophages
*Tg(ET30*:*lyzC:DsRed)* ^(^ ^30^ ^)^	*lyzC*:DsRed	Neutrophils

### *wnt16* CRISPR mutant zebrafish

gRNAs were designed targeting exon 2 of *wnt16. g*RNAs were incubated with Cas9 protein (B25641; Thermo Fisher Scientific) before injections that were performed on one‐cell‐stage eggs. CRISPR/Cas9 mutagenesis was used to generate G0 mosaic zebrafish carrying indel mutations in exon 2 of *wnt16* as previously described in Brunt *et al*.,.^(^ ^31^ ^)^ G0s were raised to 3 months and crossed to WT fish (TL/EKK strain) to generate heterozygous G1 embryos with a variety of *wnt16* mutant alleles. DNA was extracted from G1s, followed by PCR and cloning using TOPO‐TA sequencing kit (Thermo Fisher Scientific), followed by sequencing. Two alleles were selected: *wnt16* ^*bi667*^ (165 bp insertion, *wnt16* ^*a1−/−*^) and *wnt16* ^*bi451*^ (72 bp insertion, *wnt16* ^*a2−/−*^; Supplementary Fig. S1 *A*). Both alleles led to a premature stop codon compromising over 85% of the protein, likely resulting in nonsense–mediated decay and therefore predicted to be null mutants (Supplementary Fig. S1 *B*). Heterozygous *wnt16* ^*+/−*^ fish were incrossed to generate stable homozygous (*wnt16* ^*−/−*^) mutants that were used in experiments. Fish were genotyped by dorsal fin clipping into base solution (25mM NaOH, 0.2mM EDTA). Samples were heated to 98°C for 30 minutes and cooled to 4°C before neutralizing with 40mM Tris–HCl (pH 5.0). PCR was performed using EmeraldAmp GT PCR Master Mix and *wnt16* F‐ TTTTCCTCGGGCCTGGTTAT; R‐ GCCCTCTTTAACGCTCGGTA primers. Gel electrophoresis was performed using the PCR product from each sample (1.5% agarose in Tris‐acetate‐EDTA + 1:10,000 SYBR Safe; Invitrogen). Genotype was determined based on band separation caused by variation in amplicon length (Supplementary Fig. S1 *C*).

### Fracture induction and imaging

Young adult fish (6 months old) were anesthetized using MS222 (Sigma‐Aldrich) and moved onto a plastic dish. Fins were imaged before injury (see below). Fractures were induced by pressing on an individual segment of bone in the caudal fin lepidotrichia with a blunt‐ended glass capillary tube. Fractures were induced proximal to the body of the fish before the first bifurcation in the ray. Fish were recovered and reimaged at various times postinjury. Fish were housed individually and placed under anesthetic at times of interest postfracture. Fractures were imaged in the dark using a DFC700T camera mounted to a MZ10F Stereomicroscope (Leica Microsystems) before fish were revived immediately in fresh system water. Images were acquired using LAS X software 3.7.0 (Leica Microsystems).

### Live‐staining of bone

Alizarin Red stain was composed of 74μM Alizarin powder (Sigma‐Aldrich) and 5mM HEPES dissolved in Danieau's solution. Calcein green stain was composed of 40μM calcein powder (Sigma‐Aldrich) dissolved in Danieau's (pH 8). Live fish were immersed in either stain for 1 hour, then in fresh system water for 15 minutes before imaging.

### *In situ* hybridization

The RNAscope Multiplex Fluorescent Reagent kit v2 (ACD; Biotechne) was used in combination with Dr‐wnt16‐C1 (894261‐C1) and Dr‐runx2a‐C2 (409521‐C2). A TSA Cyanine 3 and 5, TMR, Fluorescein Evaluation kit (NEL760001KT; PerkinElmer) was used for staining. Briefly, fins were fixed in 4% paraformaldehyde (PFA) for 2 hours at room temperature, washed, and dehydrated in a series of increasing methanol (MeOH) concentrations. All MeOH was removed and fins were air‐dried for 30 minutes. Fins were digested in Pretreat Plus (ACD; Biotechne) for 45 minutes at room temperature and washed. RNAscope assay was performed according to the manufacturer's instructions. Some samples underwent immunohistochemistry staining before imaging by confocal microscopy.

### Whole‐mount fin immunohistochemistry

Whole fins were amputated and fixed in 4% PFA overnight at 4°C. Fins were dehydrated in a series of increasing concentrations up to 100% MeOH and stored at −20°C. Fins were rehydrated and then washed three times in PBS‐Tx (0.02% Triton‐X in PBS) for 10 minutes before permeabilization in PBS‐Tx + proteinase K (1:1000; P5568; Sigma‐Aldrich) at 37°C for 90 minutes. Solutions were refreshed every 30 minutes. Samples were washed three times in PBS‐Tx for 10 minutes, and then blocked for 3 hours in blocking buffer (5% horse serum in PBS) and incubated in primary antibody overnight at 4°C. Samples were washed in PBS‐Tx and blocked for 2 hours in blocking buffer staining with secondary antibody for 2 hours. Primary antibodies were mAb to GFP (1:500; ab13970; Abcam) and Col2a1 (1:50; M3F7; Developmental Studies Hybridoma Bank [DSHB]). Secondary antibodies were Alexa Fluor‐568 and Alexa Fluor‐488 (Thermo Fisher Scientific). Steps were performed at room temperature unless stated otherwise. Samples were mounted laterally in 1% agarose and imaged with a ×10 objective lens on a SP5 confocal microscope (Leica Microsystems).

### Whole‐mount larval immunohistochemistry

Larvae were euthanized in MS222 and fixed and dehydrated as described above. Larvae were rehydrated, then washed in PBS‐Tw (0.1% TWEEN‐20 in PBS) and permeabilized in PBS‐Tw + proteinase K (1:1000) at 37°C for 25 minutes (3 dpf) or 50 minutes (5 dpf), with solutions refreshed after 30 minutes. Samples were washed in PBS‐Tw and then blocked for 3 hours in blocking buffer before being stained and imaged as above. Larvae were mounted and imaged ventrally. Primary antibodies were chick α‐L‐plastin (gift from the Martin laboratory^(^ ^32^ ^)^) and Col2a1 (1:50; M3F7; DSHB). Secondary antibodies were Alexa‐488, DyLight 550 (Thermo Fisher Scientific).

### Alcian Blue staining

Fins were fixed in 4% PFA as previously described and dehydrated in 50%, then 70% EtOH, for 30 minutes each. Fins were stained overnight at room temperature in Alcian Blue solution composed of 0.02% Alcian Blue (Sigma‐Aldrich), magnesium chloride (60mM), and 95% EtOH. Fins were washed three times for 10 minutes each with 0.5% potassium hydroxide (KOH) and bleached for 90 minutes at room temperature in solution containing 0.5% KOH and 3% H_2_O_2_. Fins were stored in 70% glycerol before imaging.

### Tartrate‐resistant acid phosphatase staining

An acid phosphatase kit was used to detect osteoclast activity (387A; Sigma‐Aldrich). Fractures were induced in WT and *wnt16* ^*−/−*^ *mpeg1*:mCherry zebrafish before being imaged and amputated at 0 hours postinjury (hpi), 24 hpi, 4 days postinjury (dpi), and 7 dpi. Amputated fins were fixed for 40 minutes at room temperature in TRAP‐fix solution, comprised of 24% citrate solution (from kit), 65% acetone, 8% formaldehyde (37%), and 3% deionized water. Samples were washed in PBS‐Tx three times. TRAP staining solution was prepared according to the kit instructions. Fins were moved to a 24‐well plate and incubated at 37°C for 2 hours in 300 mL of TRAP stain. Fins were washed three times in PSB‐Tx and postfixed for 40 minutes at room temperature in 4% PFA before being transferred into 75% glycerol. Fins were stored at 4°C before imaging on a stereomicroscope.

### Micro‐computed tomography

Adult fish were fixed in 4% PFA for 1 week followed by sequential dehydration to 70% ethanol. Fish were scanned using a Bruker SKYSCAN 1227 μCT scanner with a voxel size of 5 μm, using an x‐ray source of 60 keV, 50 W current, and a 0.25‐mm‐thick aluminum filter. Each scan acquired 1500 angular projections with 400‐ms exposure time over a 180‐degree scan. X‐radiographs were reconstructed using the filtered backprojection algorithm provided by NRecon software (version 1.7.1.0; Bruker) and saved as 8‐bit tiff stacks. “Phantom” samples of known hydroxyapatite concentrations (0.25 and 0.75 g/cm^−3^ calcium hydroxyapatite) were also scanned using identical settings to calibrate estimates of BMD in the μCT fin data. Avizo image analysis software (version 8.0; Thermo Fisher Scientific) was used to generate 3D volume renders of whole fins using a combination of automatic and manual segmentation, which were saved as binary image stacks. The first two dorsal and ventral lepidotrichia were excluded from the analysis of all fins because of varying resolution. Image stacks were used to isolate the grayscale values of segmented fins from values of surrounding soft tissue and air by multiplying these binary (fin = 1; nonfin = 0) stacks against the original reconstruction stacks using image algebra in Fiji/ImageJ.^(^ ^33^ ^)^ Grayscale values within resulting stacks, where values >0 consisted solely of those representing fins, were compared with the mean grayscale values of both phantoms to calibrate the TMD values that they represent.

### Fluorescent image analysis

To quantify relative fluorescence intensities in fractures within transgenic fish, FIJI was used. The average intensity for each fracture within a region of interest (ROI) was measured and divided by the average intensity of uninjured bone in the same fish to give an “intensity ratio”; this analysis method normalizes for variability of reporter expression between fish and allows for standardized comparison between individuals.

$$\text{Intensity ratio}=\frac{\text{Average intensity of}\;x\;\text{within}\;\mathrm{ROI}\;\mathrm{at}\;\text{fracture site}}{\text{Average intensity of}\;x\;\text{in uninjured bone in the same fish}}$$

x=stain or transgene reporter of interest,suchaseGFP

To analyze the number of immune cells responding to fracture, we used the freely available Modular Image Analysis (MIA; version 0.9.30) workflow automation plugin for Fiji.^(^ ^34^, ^35^ ^)^ Images were enhanced using the WEKA pixel classification plugin^(^ ^36^ ^)^ and thresholded at a probability of 0.5. Adjacent cells in the binarized image were separated using an intensity‐based watershed transform and individual cells subsequently identified as regions of connected foreground‐labeled pixels.^(^ ^37^ ^)^ Cells were subjected to a size filter, retaining only those in the range 30 to 500 μm^2^. The distance of each cell to the manually identified fracture was measured.

### Statistical analysis

Statistical analyses were performed, and graphs were created in GraphPad PRISM 8 software. Where possible, a D'Agostino Pearson normality test was performed on data to determine whether a parametric or nonparametric statistical test should be used. Where two or more data sets were compared, a one‐way analysis of variance (ANOVA) or a Kruskal‐Wallis test was used to determine statistically significant differences between groups for parametric and nonparametric data, respectively. For comparison of WT and *wnt16* mutants throughout fracture repair, multiple *t* tests were performed at each time point using the Holm‐Sidak correction to calculate *p* values. Differences were considered statistically significant where *p* < 0.05.

## Results

### Young *wnt16* mutant zebrafish are susceptible to spontaneous fractures that heal more slowly compared with WT fish

**Fig 1:**
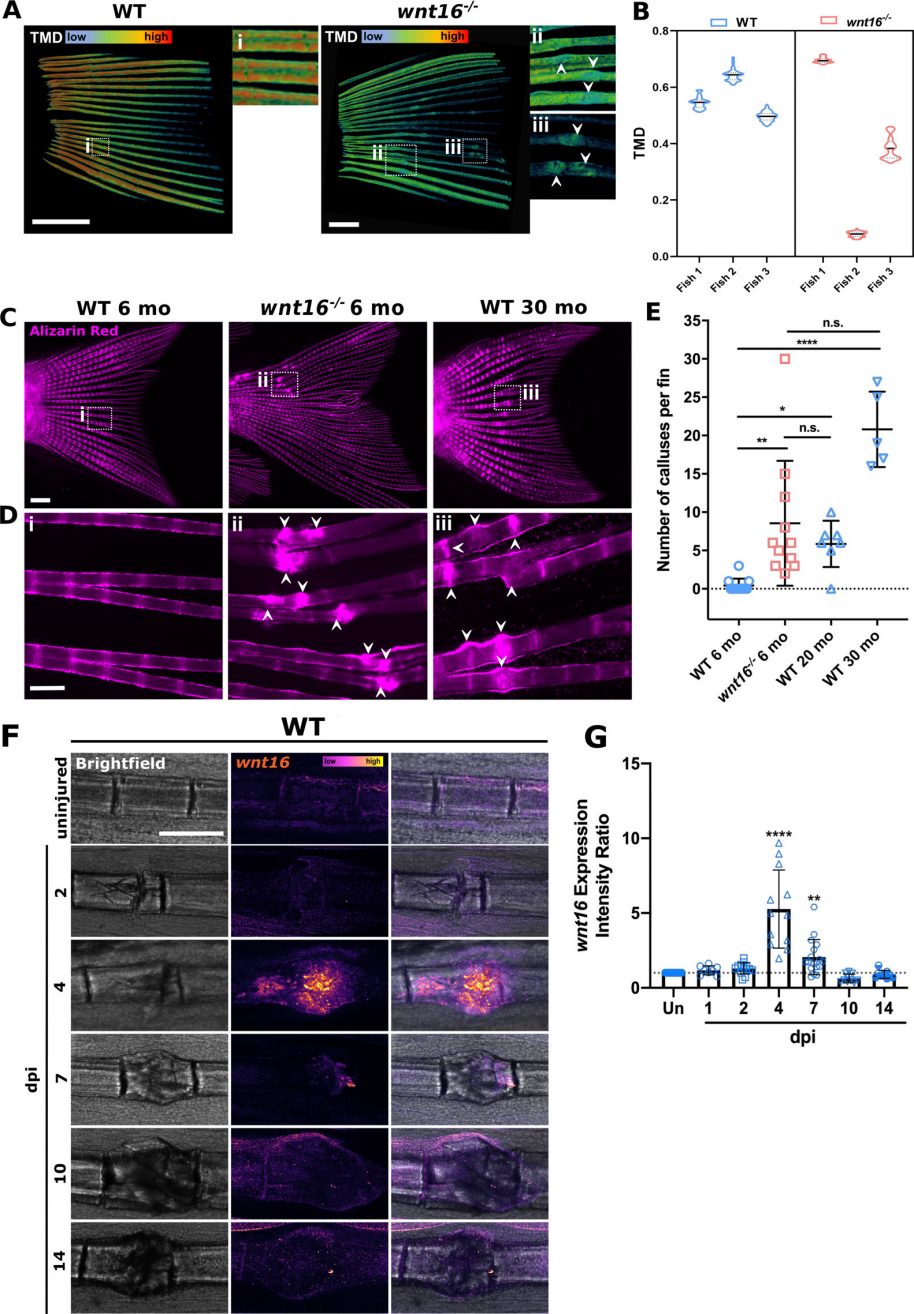
The *Wnt16* mutants are susceptible to spontaneous fractures while *wnt16* expression is upregulated in injured bone. (*A*) μCT images indicate lower and more variable tissue mineral density (TMD) and the presence of bone calluses (arrowheads) in the fins of *wnt16* ^*−/−*^ zebrafish. (*B*) Violin plots show distribution around mean (black line) TMD in WT and *wnt16* ^*−/−*^ fins. N = 3; scale bar = 1 mm. (*C*) Uninjured WT and *wnt16* ^*−/−*^ zebrafish were live‐stained with Alizarin Red at ages 6, 20, or 30 months (mo). Scale = 1 mm. (*D*) Higher magnification of fins (from *C*) shows the presence of bone calluses (arrowheads) resulting from bone repair in 6‐month‐old *wnt16* ^*−/−*^ and 30‐month‐old WT zebrafish but not 6‐month‐old zebrafish. Scale = 200 μm. (*E*) Quantification of bone calluses per fin shows that young *wnt16* mutants display a significantly higher number of calluses compared with WT fish at the same age but no significant difference compared with aged WT zebrafish. N ≥ 5 per condition. (*F*) Representative images showing fluorescent *in situ* hybridization of *wnt16* performed on WT uninjured bone and fractures between 2 and 14 dpi. (*G*) *wnt16* expression within the fracture site was quantified relative to uninjured bone (un) in the same fin (intensity ratio). The expression of *wnt16* increased significantly postfracture between 4 and 7 dpi. *p < 0.05, ****p < 0.0001; N ≥ 8 per time point.

*WNT16* has been associated with low eBMD and increased fracture risk.^(^ ^12^, ^14^, ^38^ ^)^ Therefore, we used μCT to observe bone morphology and TMD in whole fins of adult WT and *wnt16* ^*−/−*^ zebrafish. The *wnt16* mutants displayed a high degree of variability in TMD relative to WT specimens, as well as lower TMD (Fig. 1*A*,*B*). Images of *wnt16* ^*−/−*^ fins showed a high number of bone calluses (Fig. 1*A*) that form postfracture and do not completely resolve after the bone has repaired.^(^ ^24^ ^)^ Bone calluses in the caudal fin rays can be easily visualized using Alizarin Red S (ARS). Thus, we next used ARS to compare the frequency of spontaneous lepidotrichia fractures in 6‐month‐old WT and 6‐month‐old *wnt16* ^*−/−*^ uninjured fish. Bone calluses and spontaneous fractures were rarely observed in the 6‐month‐old WT fish, with only 25% of fish sampled displaying a minimal number of calluses (≤ 3; Fig. 1*C‐E*). However, a significantly higher number of calluses were recorded in 6‐month‐old *wnt16* ^*−/−*^ fins; 100% of *wnt16* ^*−/−*^ fins sampled contained calluses, with a mean of 8.5 calluses per fin versus 0.4 calluses per fin in WT fish. To test whether callus quantity increases with age, we quantified callus number in 20‐month‐old and 30‐month‐old WT fish. Aged WT fish were comparable in appearance and callus frequency to 6‐month‐old *wnt16* ^*−/−*^ fish (Fig. 1*E*). Collectively, this shows that *wnt16* ^*−/−*^ fish display a bone fragility phenotype predisposing them to spontaneous fractures and the accumulation of calluses at a young age.

### *wnt16* expression is significantly upregulated in bone postfracture

Fracture repair in zebrafish commences with the recruitment of immune cells before osteoblast activity later increases to facilitate bone callus mineralization.^(^ ^24^ ^)^ Because WNT16 has been linked to immune cell differentiation and osteoblast function,^(^ ^15^, ^18^ ^)^ we next sought to establish whether *wnt16* was expressed during fracture repair in zebrafish. Fractures were induced on a bone segment within the caudal fin lepidotrichia of 6‐month‐old WT zebrafish. Using RNAscope, whole‐mount *in situ* hybridization was performed on fins fixed between 1 and 14 dpi (Fig. 1*F*). *wnt16* was expressed at low levels in uninjured bone, but expression increased significantly at 4 dpi, before returning to basal levels by 10 dpi (Fig. 1*G*). This shows that *wnt16* expression is upregulated early on postfracture, suggesting a role for Wnt16 in the initiation of bone repair.

### Bone mineralization but not soft callus formation is delayed postfracture in *wnt16* mutants

**Fig 2:**
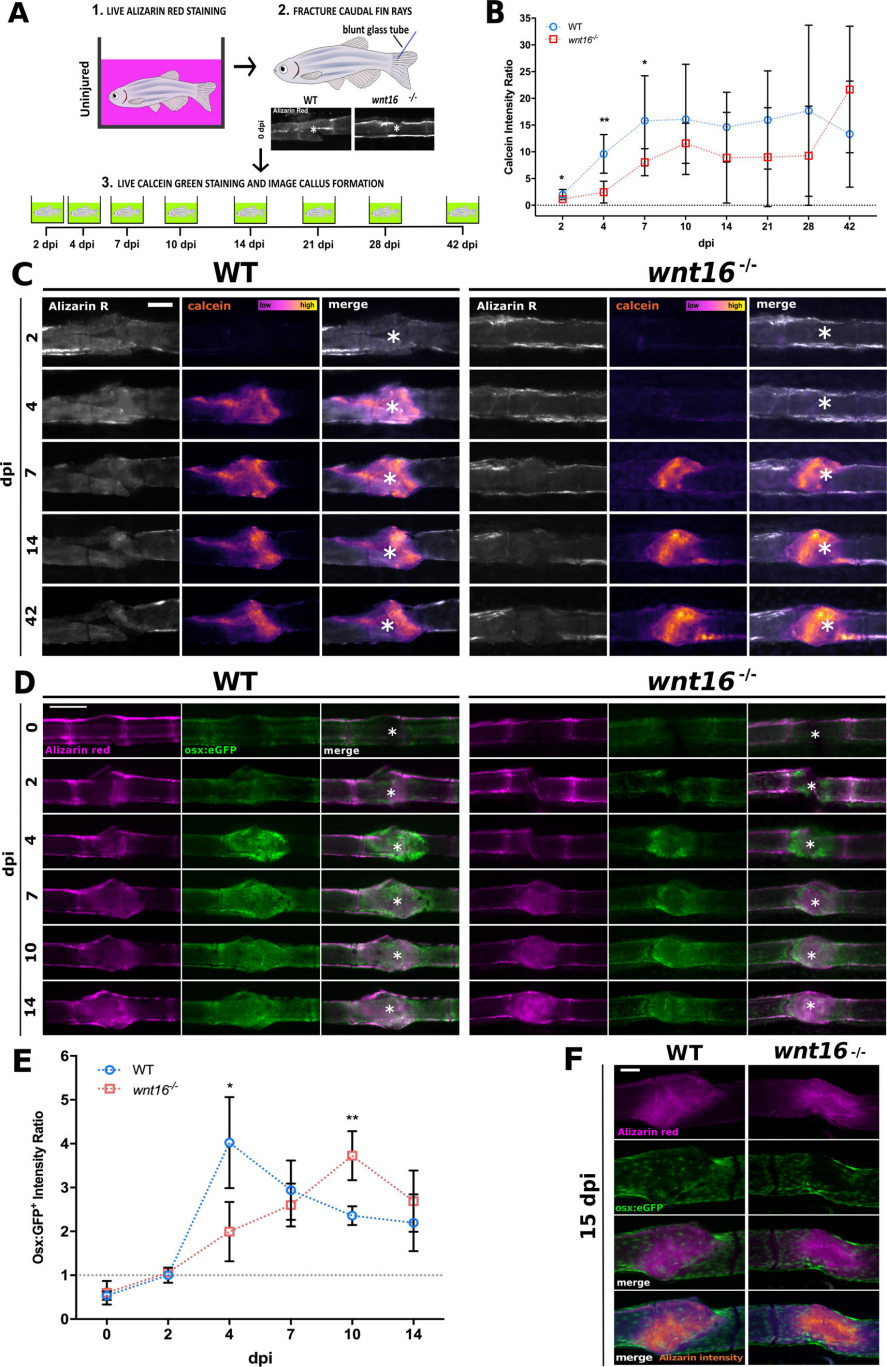
Bone mineralization and osteoblast recruitment is significantly delayed postfracture in *wnt16* ^*−/−*^ zebrafish. (*A*) Schematic illustrating fracture induction assay and labeling of old bone (Alizarin Red) and new bone (calcein green). (*B*) Callus formation was quantified by measuring the calcein intensity ratio between the fracture site and uninjured bone. Callus formation was significantly reduced from 2 to 7 days postinjury (dpi) in *wnt16* mutant compared with WT fractures. N ≥ 5 per condition. Gray dotted line indicates where calcein intensity at the fracture site = uninjured bone. (*C*) Representative images of WT and *wnt16* ^*−/−*^ fish at selected time points postinjury show old bone labeled by Alizarin Red (gray) and callus formation labeled by calcein. White asterisk = center of fracture. Scale = 200 μm. (*D*) Representative images of calcified bone (Alizarin Red) and osteoblasts (*osx*:GFP) at fracture site in WT and *wnt16* ^*−/−*^ throughout fracture repair. White asterisk = center of fracture. Scale bar = 100 μm. (*E*) Osteoblast density was quantified by measuring the fluorescence intensity of *osx*:GFP within the fracture site normalized to control bone in the same fin (intensity ratio). Gray dotted line indicates where *osx*:GFP intensity at the fracture site = uninjured bone. Osteoblast recruitment was delayed in *wnt16* mutants, which had a significantly lower *osx*:GFP intensity ratio at the fracture site 4 dpi, but significantly higher *osx*:GFP intensity ratio at 10 dpi compared with WT zebrafish. (*F*) Confocal imaging of bone in amputated fins at the end of the time course (15 dpi) shows complete union of fractures in both WT and *wnt16* ^*−/−*^ zebrafish. Scale bar = 100 μm. (*B* & *E*): N.s = no significant difference, *p < 0.05, **p < 0.01, ****p < 0.0001. N ≥ 6 per genotype.

Because *wnt16* is expression is upregulated postfracture and *wnt16* mutants displayed a high number of bone calluses, we next tested whether fracture repair was impaired in *wnt16* ^*−/−*^ zebrafish. Adult WT and *wnt16* ^*−/−*^ zebrafish were live‐stained in ARS to label bone and imaged before fracture induction. Zebrafish were then live‐stained in calcein green to label newly incorporated bone matrix at the fracture site, which was reimaged at the time points indicated (Fig. 2*A*). Injured *wnt16* ^*−/−*^ zebrafish displayed significantly reduced bone callus formation within the first 7 days of fracture healing compared with WT fish, which was most apparent at 4 dpi (Fig. 2*B*,*C*). We also investigated whether formation of the initial soft callus, typically comprised of glycosaminoglycan‐rich cartilaginous matrix,^(^ ^39^ ^)^ differed between WT and *wnt16* mutant zebrafish. Alcian Blue staining showed the presence of a cartilaginous soft callus, peaking at 4 dpi (Supplementary Fig. S2 *A*). However, no difference in Alcian Blue staining was observed between WT and *wnt16* mutant bone postfracture. Fractures were also induced in the caudal fins of transgenic *col2a1*:mCherry zebrafish (Table 1) to observe chondrocyte activity postfracture. mCherry expression was almost undetectable throughout fracture repair, and intensity ratios showed little variation from uninjured bone at all time points postinjury (Supplementary Fig. S2 *B*,*C*). Moreover, no significant differences in Col2a1 levels were observed between WT and *wnt16* mutant fractures at any time point. The transgenic data were validated using immunohistochemistry for Col2a1 at 4 dpi on fixed WT fins. No observable increase in Col2a1 was detected at 4 dpi, relative to uninjured bone (Fig. Supplementary S2 *D*). Collectively, these data suggest that soft callus formation is not affected by loss of *wnt16*, and that Col2a1 is not a predominant component of the soft callus formed postfracture in zebrafish lepidotrichia.

### Osteoblast recruitment is delayed in *wnt16* ^−/−^ zebrafish postfracture

Osteoblast activation is a key event in the bone repair process postfracture. Osteoblasts differentiate from mesenchymal stem cell (MSC) precursors, initially expressing *runx2* before downregulating *runx2* and expressing the transcription factor osterix (*osx*). *osx* ^*+*^ osteoblasts synthesize bone matrix within the initial soft callus; the callus hardens as it mineralizes and is remodeled to restore the bone to a healthy state.^(^ ^40^ ^)^ Moreover, transcriptomic analysis of osteoblast‐prone clones isolated from tonsil–derived MSCs showed that upregulation of WNT16 is predictive of osteogenic differentiation.^(^ ^41^ ^)^ In zebrafish, osteoblasts dedifferentiate and proliferate in response to bone injury, migrating to the damaged tissue where they initiate bone repair.^(^ ^42^ ^)^ Thus, we next investigated whether osteoblast activity impaired postfracture repair in *wnt16* ^*−/−*^ zebrafish. We performed live ARS before fin fractures of WT and *wnt16* ^*−/−*^ zebrafish carrying the osteoblast‐labeling transgene, *osx*:GFP (Table 1). Fractures were induced and restained with live ARS before imaging to ensure labeling of any new bone. The intensity of *osx*:GFP signal was measured as a ratio between the fracture site and uninjured bone to quantify osteoblast recruitment throughout fracture repair (0–14 dpi). In WT zebrafish, the relative intensity of *osx*:GFP at the fracture site peaked rapidly at 4 dpi before steadily decreasing (Fig. 2*D*,*E*). However, the relative intensity of *osx*:GFP was significantly reduced at 4 dpi in *wnt16* mutants, not peaking until 10 dpi (Fig. 2*D*,*E*). A comparable bony callus had formed at the fracture site in both WT and *wnt16* ^*−/−*^ by 15 dpi (Fig. 2*F*). This shows that osteoblasts in *wnt16* ^*−/−*^ zebrafish can respond to bone injury but that the recruitment and activity of these osteoblasts are significantly delayed. Reduced osteoblast activity at 4 dpi in *wnt16* mutants coincided with the peak of *wnt16* expression postfracture in WT bone (Fig. 1*E*,*F*) and delayed mineralization in *wnt16* ^*−/−*^ fractures (Fig. 2*A*,*B*), suggesting that *wnt16* is required for the initiation of optimal bone repair.

### Innate immune cell dynamics are unaltered in *wnt16* ^−/−^ zebrafish postfracture

**Fig 3:**
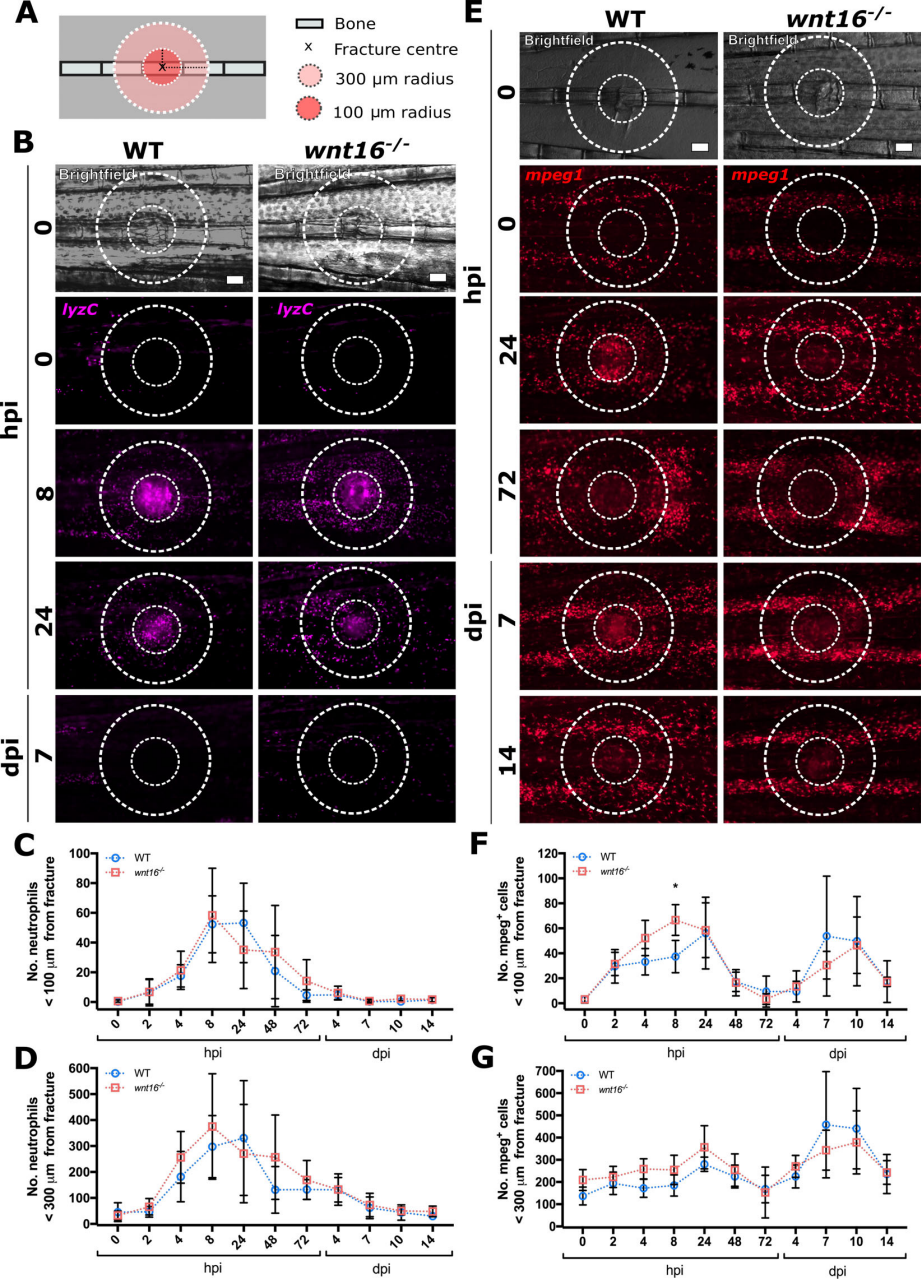
Loss of Wnt16 does not perturb leukocyte recruitment to bone postfracture. Fractures were induced in WT and *wnt16* ^*−/−*^ zebrafish carrying *lyzC*:DsRed and *mpeg1*:mCherry transgenes to measure the recruitment of neutrophils and macrophages, respectively. (*A*) Schematic depicting regions of interest around the fracture site where leukocyte recruitment was quantified. (*B*) Representative images of from WT and *wnt16* ^*−/−*^ zebrafish show neutrophil (*lyzC* ^+^ cells) recruitment to fractured bone at 0, 8, and 24 hours postinjury (hpi) and 7 days postinjury (dpi). Scale bar = 100 μm. (*C*,*D*) The number of neutrophils within 100 μm (*C*) and 300 μm (*D*) of the fractures were quantified in an automated manner using modular image analysis (MIA) from 0 hpi to 14 dpi. WT and wnt16 mutants displayed comparable numbers of neutrophils at the fracture site at all time points post injury. N ≥ 5 per genotype. (*E*) Representative images of from WT and *wnt16* ^*−/−*^ zebrafish show macrophage (*mpeg1* ^+^ cells) recruitment to fractured bone at selected time points from 0 to 14 dpi. Scale bar = 100 μm. (*F*,*G*) The number of neutrophils within 100 μm (*F*) and 300 μm (*G*) of the fractures were quantified using MIA from 0 hpi to 14 dpi. WT and wnt16 mutants displayed comparable numbers of macrophages at all time points postinjury, with the exception of 8 hpi when *wnt16* mutants had recruited significantly more macrophages to within 100 μm of the fracture site (*F*). *p < 0.05; N ≥ 5 per genotype.

**Fig 4:**
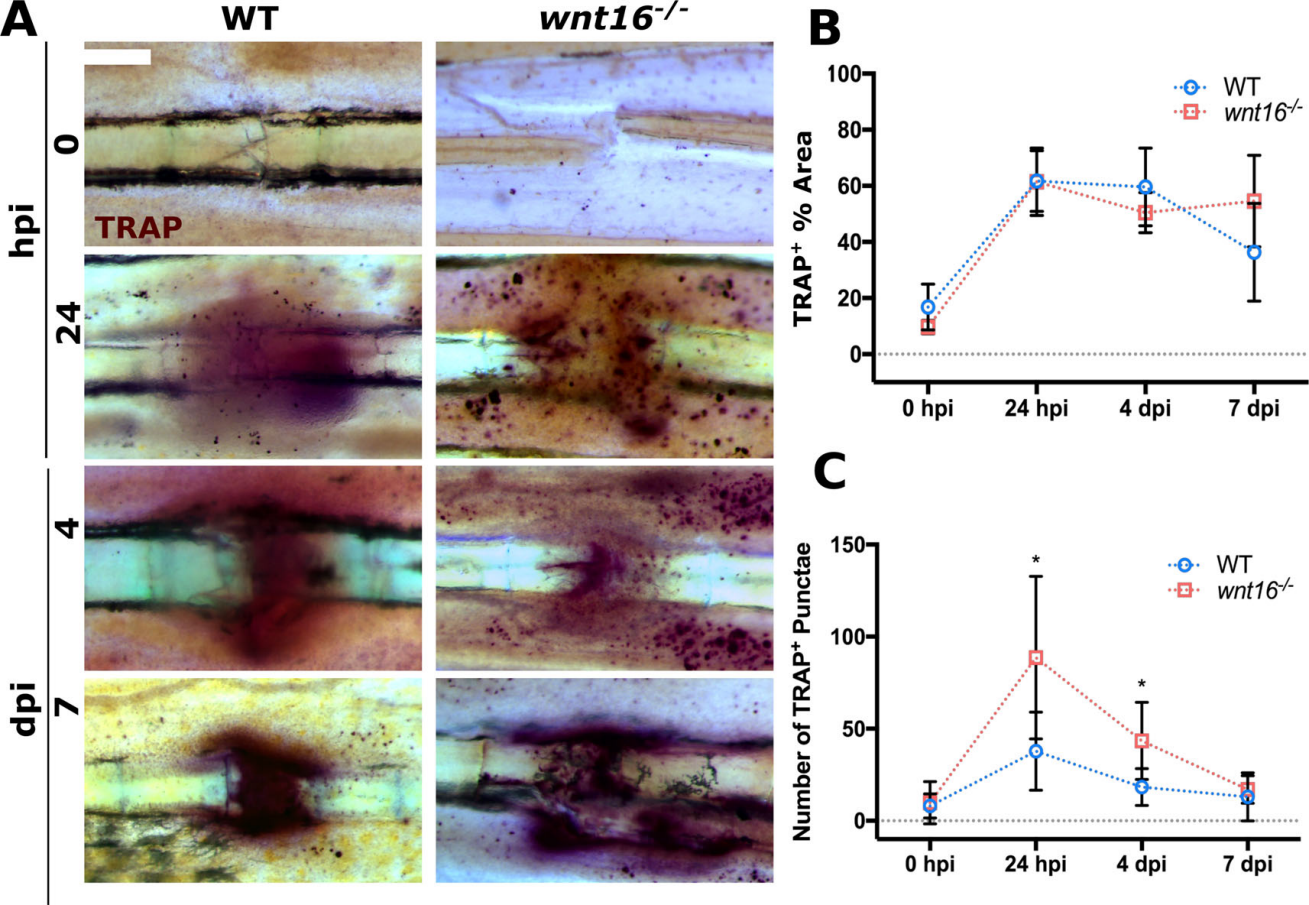
TRAP^+^ punctae accumulate near to fractures in *wnt16* ^*−/−*^ zebrafish postinjury. Fins from WT and *wnt16* ^*−/−*^ zebrafish were amputated at 0 hours postfracture (hpi), 24 hpi, 4 days postfracture (dpi) and 7 dpi before undergoing staining to detect the presence of tartrate‐resistant acid phosphatase (TRAP). (*A*) Representative images of fractures stained for TRAP. Scale bar = 100 μm. (*B*) Overall coverage of TRAP was measured by calculating the total % area stained within 300 μm of the fracture site. No significant difference in the amount of TRAP^+^ stained area between WT and *wnt16* ^*−/−*^ fractures was found. (*C*) The number of TRAP^+^ punctae present within 300 μm of the fracture site were quantified and showed a significantly higher number of punctae at 24 hpi and 4 dpi in the fractures of *wnt16* ^*−/−*^ zebrafish compared with WT. *p < 0.05; N ≥ 6 per genotype.

Fracture repair has been shown to comprise an inflammatory phase, a repair phase, and a remodeling phase in mammals.^(^ ^43^ ^)^ The controlled recruitment, activity, and reverse migration of leukocytes during the inflammatory phase are known to be prerequisites for initiating osteoblast activity and optimal bone repair.^(^ ^44^ ^)^ Neutrophils are among the first cells to be recruited to fractures.^(21)^ Stimulation of noncanonical Wnt pathways with recombinant WNT5a has been shown to initiate chemotactic migration and chemokine production in neutrophils, but whether WNT16 influences neutrophil recruitment is unknown.^(^ ^45^ ^)^ Macrophages also rapidly respond to bone damage and continue to aid throughout the repair and remodeling phases in mammalian models of fracture.^(^ ^20^ ^)^ A previous study indicated that *wnt16* expression was required for functional hematopoiesis in zebrafish embryos.^(^ ^18^ ^)^ Additionally, overexpression of *WNT16* in mouse osteoblast‐progenitor cells has been shown to partially rescue glucocorticoid‐induced osteoporosis,^(^ ^46^ ^)^ suggesting that Wnt16 may regulate osteoblast activity and bone repair via immune cells. To validate whether early leukocyte development was impaired in *wnt16* mutants, we fixed zebrafish larvae at 3 and 5 dpf. Whole‐mount immunohistochemistry was used to label cartilage in the developing skeleton (Col2a1) and immune cells (L‐plastin), but surprisingly no differences in leukocyte numbers were observed at either age (Supplementary Fig. S3). Despite this, because early callus formation and osteoblast differentiation were delayed in *wnt16* ^*−/−*^ fractures, we also investigated whether immune cell recruitment to bone injury was altered in adult *wnt16* mutants. To address this, we used *lyzC*:DsRed (neutrophils) and *mpeg1*:mCherry (macrophages) transgenic zebrafish lines (Table 1) to study leukocyte dynamics postfracture in WT and *wnt16* ^*−/−*^ zebrafish. Immune cell recruitment relative to the fracture site over time was quantified using modular image analysis.^(^ ^34^ ^)^ The number of neutrophils (*lyzC* ^+^ cells) and macrophages (*mpeg1* ^+^ cells) within a 100‐μm radius and 300‐μm radius of the fracture were calculated (Fig. 3*A*). In both WT and *wnt16* ^*−/−*^ zebrafish, neutrophils were rapidly recruited to the fracture, peaking between 8 and 24 hpi (Fig. 3*B*). No significant differences in the number of neutrophils recruited to the fracture sites of WT and *wnt16* ^*−/−*^ zebrafish were detected at any time point postinjury (Fig. 3*C*,*D*). Macrophages were also rapidly recruited to fractures in the first 24 hpi (Fig. 3*E*). Interestingly, we observed that macrophages responded to fracture in a biphasic manner, decreasing in number from 2 to 4 dpi, before peaking in number for a second time at approximately 7 dpi (Fig. 3*F*,*G*). This suggests that phenotypically distinct populations of macrophages may be required at different stages postfracture to contribute to efficient bone repair. Comparison between WT and *wnt16* ^*−/−*^ zebrafish showed no difference in the number of *mpeg1* ^+^ cells recruited to the fracture throughout repair, aside from a significant increase in macrophage number in *wnt16* ^*−/−*^ zebrafish at 8 hpi (Fig. 4*F*,*G*). These data suggest that overall, leukocyte recruitment to fractures is not impaired in *wnt16* mutants and does not contribute to delayed bone repair resulting from loss of Wnt16.

### Patterning of TRAP activity is altered in *wnt16* ^−/−^ zebrafish

TRAP‐synthesizing osteoclasts are required to resorb damaged bone but must be regulated to prevent osteoporosis.^(^ ^47^ ^)^ Recombinant WNT16 has been shown to suppress osteoclastogenesis and TRAP activity *in vitro* by regulating osteoprotegerin expression in osteoblasts.^(^ ^48^ ^)^ The uptake of osteoblast‐derived extracellular vesicles by immature osteoclasts has been shown to promote osteoclast differentiation in zebrafish scale fractures, confirming that intercellular communication between osteoblasts and osteoclasts regulates osteoclastogenesis in response to bone damage.^(^ ^49^ ^)^ Osteoclasts and macrophages are derived from a common myeloid lineage, with peripheral blood monocytes showing higher osteoclastic potential compared with bone marrow–derived monocytes.^(^ ^50^ ^)^ Moreover, a previous study established that cells expressing the osteoclast marker cathepsin K infiltrate the lepidotrichia fracture site where TRAP is detected by 24 hpi in zebrafish^(^ ^24^ ^)^; this coincides with the recruitment of the initial wave of *mpeg1*–expressing cells to the fracture site observed in our model (Fig. 3*E*‐*G*). Therefore, we investigated whether TRAP activity postfracture was associated with the recruitment of *mpeg* ^*+*^ cells and whether loss of Wnt16 affected levels of TRAP. Fractures were induced in *mpeg1*:mCherry^+^ WT and *wnt16* ^*−/−*^ zebrafish and live‐imaged before amputation of the fin for TRAP staining. The overall levels of osteoclast activity were measured by calculating the percentage area of TRAP^+^‐stained tissue within a 300‐μm radius of the fracture site. Osteoclast activity increased rapidly at 24 hpi and remained high before gradually decreasing by 7 dpi (Fig. 4*A*,*B*). No significant difference in overall levels of osteoclast activity at the fracture site (TRAP^+^ % area) was detected between WT and *wnt16* ^*−/−*^ fractures (Fig. 4*B*). However, the overall patterning of TRAP staining was altered at 24 hpi and 4 dpi; *wnt16* ^*−/−*^ zebrafish displayed a significantly higher number of TRAP^+^ punctae around the fracture, whereas WT fractures tended to display fewer punctae, with continuous diffuse areas of TRAP^+^ tissue (Fig. 4*A*,*C*). Comparable patterning of TRAP^+^ punctae was not observed in uninjured bone from either WT or *wnt16* mutants. Interestingly, we observed similarities in the patterning of TRAP^+^ punctae and *mpeg1* ^+^ cells, with punctae colocalizing with *mpeg1* ^*+*^ expression in some regions (Supplementary Fig. S4). This suggests that *mpeg1*‐expressing cells may contribute to bone remodeling and TRAP‐synthesis during the early stages of fracture repair.

### Precocious activation of the canonical Wnt signaling pathway may underpin delayed bone repair in *wnt16* ^−/−^ zebrafish postfracture

**Fig 5:**
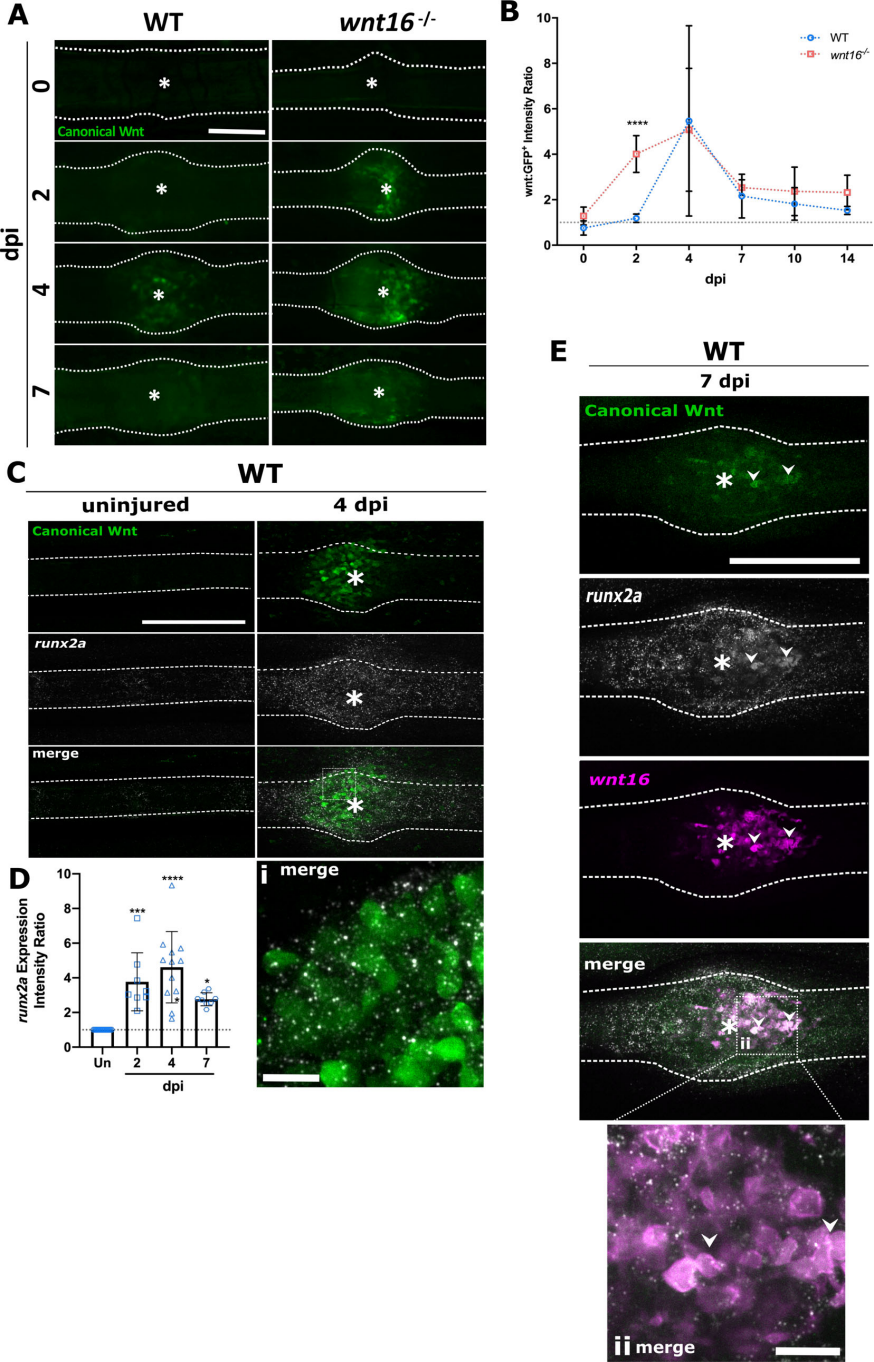
The *wnt16* ^*−/−*^ zebrafish display precocious activation of the canonical Wnt pathway during preosteoblast proliferation and differentiation postfracture. (*A*) Fractures were induced in Wnt:GFP transgenic zebrafish that express GFP in cells responding to activation of the canonical Wnt signaling pathway. Representative images are shown from 0 to 7 days postfracture (dpi). (*B*) Levels of Wnt pathway activation throughout fracture repair were quantified by measuring the fluorescence intensity of Wnt:GFP within the fracture site normalized to control bone in the same fin (intensity ratio). Gray dotted line indicates where canonical Wnt activity at the fracture site = uninjured bone. The *wnt16* ^*−/−*^ zebrafish displayed significantly higher levels of canonical Wnt activity at 2 dpi compared with WT fractures. High levels of Wnt:GFP at the fracture site were sustained through to 4 dpi in *wnt16* mutants, where they became comparable with WT. n ≥ 6 per genotype. (*C*) *In situ* hybridization of *runx2a* in WT uninjured and fractured fins at 4 dpi shows coexpression of *runx2a* and Wnt:GFP, both peaking at 4 dpi (i). (*D*) Expression of *runx2a* (measured as above) increased significantly by 2 dpi, peaking at 4 dpi, before decreasing at 7 dpi. Un = Uninjured control, n ≥ 8 per time point. (*E*) *In situ* hybridization of fractures at 7 dpi showed the colocalization of *runx2a*, *wnt16* with low levels of Wnt:GFP (ii). ****p < 0.0001, ***p < 0.001, *p < 0.05. Dotted lines = bone outline; white asterisk = center of fracture. Scale bar *C*, *D* = 200 μm; scale bar i, ii = 20 μm.

Wnt‐signaling proteins regulate the stemness, differentiation, and proliferation of MSCs and osteoblasts. Moreover, previous studies in mice have indicated that Wnt16 may buffer levels of canonical Wnt signaling in response to injury.^(^ ^10^ ^)^ Therefore, we investigated levels of canonical Wnt activity in *wnt16* ^*−/−*^ zebrafish postfracture using a β‐catenin–responsive transgenic line (Wnt:GFP; Table 1). Fractures were induced in the caudal lepidotrichia of the fish and imaged at identical time points as in Fig. 2*E*. In *wnt16* ^*−/−*^ zebrafish, we observed a significant increase in the intensity ratio of canonical Wnt‐responsive cells at the fracture site from 2 dpi compared with WT zebrafish (Fig. 5). Canonical Wnt signaling remained elevated in *wnt16* ^*−/−*^ fractures through to 4 dpi, where Wnt:GFP intensity ratios were comparable with WT fractures, before gradually decreasing to homeostatic levels by 10 dpi (Fig. 5*B*). This suggests that enhanced canonical Wnt signaling may contribute toward delayed callus formation and osteoblast differentiation in response to fracture in *wnt16* ^*−/−*^ zebrafish. However, precocious canonical Wnt activation occurs at 2 dpi in *wnt16* mutants prior to when *wnt16* expression is normally upregulated postfracture (4 dpi). Hence, it is plausible that the loss of Wnt16 influences canonical Wnt activity indirectly by governing the differentiation of proliferating preosteoblasts into osteoblasts. Runx2 is a transcription factor that is strongly expressed by osteoblast precursors and is required for the proliferation of preosteoblasts.^(^ ^51^ ^)^ Runx2 directly increases the expression of canonical Wnt pathway genes such as *Tcf7*, while reciprocal signaling between canonical Wnt pathway genes and *runx2* induces the commitment of mesenchymal cells into osteoblasts.^(^ ^52^ ^)^ Therefore, we next sought to characterize the spatiotemporal dynamics of *runx2a* expression relative to canonical Wnt pathway activation and *wnt16* expression during fracture repair in WT zebrafish. Using RNAscope, we performed whole‐mount *in situ* hybridization on fins from 2 to 7 dpi. Expression of *runx2a* increased significantly relative to uninjured bone between 2 and 7 dpi, peaking at 4 dpi (Fig. 5*C*,*D*). The peak in *runx2a* expression coincided with the height of canonical Wnt activity and *wnt16* expression within the fracture site (Fig. 5*A*,*B*, Fig. 1*F*,*G*, respectively), suggesting that cells responding to canonical Wnt pathway activation may be proliferative osteoblast precursors. Indeed, at 7 dpi, as Wnt:GFP levels decreased, we detected the merged expression of *wnt16*, *runx2a*, and Wnt:GFP (Fig. 5*E*), This suggests that *wnt16* promotes the differentiation of osteoblast progenitor cells into osteoblasts.

## Discussion

Multiple studies have associated mutations in *WNT16* with osteoporosis and fracture susceptibility phenotypes in humans,^(^ ^121‐4^ ^)^ but less is known about the pathophysiological influence of *WNT16* on bone during fracture repair. Moreover, models to study the influence of GWAS–derived fracture‐susceptible candidate genes on bone dynamically *in vivo* were lacking. In this study, we found that loss of Wnt16 in zebrafish leads to variable TMD and the accumulation of bone calluses within lepidotrichia resulting from fractures at an early age. Induction of fractures in caudal fin lepidotrichia showed that *wnt16* expression is significantly upregulated between 4 and 7 dpi and that Wnt16 is required for optimal fracture repair and the rapid recruitment of osteoblasts postinjury. Alcian Blue staining showed that soft callus formation was unperturbed in *wnt16* mutants, nor was the development of leukocytes or the responsiveness of neutrophils and macrophages to bone injury. However, loss of Wnt16 altered the patterning of TRAP activity at the fracture site. We revealed that delayed fracture repair coincided with precocious activation of the canonical Wnt signaling pathway in *wnt16* mutants at 2 dpi. In WT fractures, we show that elevated expression of *runx2a* and canonical Wnt activity both peaked at 4 dpi, before colocalizing with *wnt16–* expressing cells at reduced levels at 7 dpi, suggesting that *wnt16* promotes the optimal differentiation of Wnt:GFP^+^ osteoblast progenitors into mature osteoblasts.

Disordered activation of the canonical Wnt signaling pathway has been linked to the pathogeneses of many age‐related diseases, including osteoporosis.^(^ ^8^ ^)^ Canonical Wnt signaling culminates in the accumulation of β‐catenin in the cell, which translocates to the nucleus where it binds to and activates the transcription factors, TCF/LEF (T‐cell factor/lymphoid–enhancing binding factor). WNT16 was found to be protective against excessive activation of canonical WNT and severe cartilage degeneration in an induced osteoarthritis murine model, suggesting that WNT16 may antagonize canonical Wnt activity postinjury.^(^ ^10^ ^)^ Although canonical Wnt signaling is required for osteogenesis, LEF‐1 is downregulated in the early stages of fracture repair during soft callus formation.^(^ ^53^ ^)^ Crucially, it has been shown that constitutive β‐catenin mediated activation of LEF‐1 represses the osteoblast transcriptional regulator, Runx2, and subsequent maturation of osteoblasts.^(^ ^54^ ^)^ Furthermore, osterix has been shown to negatively regulate canonical Wnt activity during osteoblast differentiation.^(^ ^55^ ^)^ In WT fractures, we observed increased expression of *runx2a* from 2 dpi continuing to 4 dpi, where both canonical Wnt activity and *runx2* expression peaked. Colocalization of Wnt:GFP and *runx2a* with *wnt16* at 7 dpi, as levels of all reduce, implies that *wnt16* promotes the suppression of canonical Wnt activity in preosteoblasts and their differentiation into osteoblasts, potentially via regulation of *runx2* and *osterix*. Collectively, this suggests that delayed callus formation postfracture in *wnt16* ^−/−^ zebrafish may be caused by the precocious and prolonged activation of the canonical Wnt signaling pathway. Precocious activation of the canonical Wnt pathway at 2 dpi may act to suppress early expression of *runx2a*, delaying the differentiation of osteoblast progenitors into mature, bone matrix–synthesizing osteoblasts. Regulation of canonical Wnt activity by *wnt16* may occur either directly or indirectly via *runx2a* and *osx*, to promote osteoblast maturation. Further studies are required to establish whether delayed bone repair in *wnt16* ^*−/−*^ zebrafish can be completely or partially rescued via pharmacological modulation of the canonical Wnt signaling pathway using Wnt inhibitor compounds such as IWR‐1.^(^ ^56^ ^)^

Morphant *wnt16* embryos have been shown to display severe impairment of hematopoiesis.^(^ ^18^ ^)^ However, we found that loss of Wnt16 had no effect on the overall number of leukocytes detected in larvae during early skeletogenesis, nor did it have an overall effect on the recruitment of neutrophils and macrophages postfracture. Evidence has shown that off‐target effects of morpholinos during gene knockdown may show more extreme phenotypes compared with stable mutant lines.^(^ ^57^ ^)^ Our data show that targeted, stable loss of Wnt16 via CRISPR‐Cas9 mutagenesis does not impair primitive hematopoiesis or the innate immune response to bone injury in adult tissues. However, further investigation into HSC line markers is required to conclusively determine whether stable mutagenesis of *wnt16* shows aberrant effects on early hematopoiesis comparable to those observed in *wnt16* morphants. One modulator of bone repair, not explored in this study, is angiogenesis. Vascularization of injured bone is crucial for the metabolically demanding process of fracture repair.^(^ ^58^ ^)^ Clements *et al.,* also showed that Wnt16 is required for somatic expression of Notch ligands^(^ ^18^ ^)^; Notch signaling is a known, central regulator of angiogenesis.^(^ ^59^ ^)^ Assessing angiogenesis postfracture using endothelial transgenic lines and measuring the expression of vascular endothelial growth factors may shed further light on the mechanisms underpinning delayed bone repair in *wnt16* mutants. However, no existing studies have shown a role for WNT16 in angiogenesis, suggesting it is unlikely that vascularization is affected in *wnt16* mutants.

Our data further support the dogma that fracture repair in zebrafish lepidotrichia has three phases, similar to mammals.^(^ ^43^ ^)^ The first is an initial inflammatory phase (~4–48 hpi), whereby neutrophils and macrophages infiltrate the fracture. This is proceeded by a repair phase (~2–10 dpi), whereby a glycosaminoglycan‐rich soft callus forms initially, before osteoblasts are activated and recruited to synthesize new bone matrix to unionize the fracture with a callus. Ultimately, the bone enters an ongoing remodeling phase (>10 dpi) in which, like humans, the repaired bone remains marked with a calcified callus. Interestingly, the biphasic recruitment of macrophages postfracture, which we observed for the first time in zebrafish, is reminiscent of mammalian bone repair. In mammals, M1‐like macrophages are observed during the inflammatory phase and replaced by reparative M2‐like macrophages, which contribute to bone matrix synthesis and the remodeling of bone.^(^ ^20^, ^50^, ^60^ ^)^ *mpeg1* has been widely used as a macrophage‐specific promoter in zebrafish transgenic lines. However, evidence has emerged from a number of recent studies showing that *mpeg1* expression is not restricted to macrophages in adult zebrafish. One study found a large proportion of *mpeg1* ^*+*^ cells to be B cells,^(^ ^61^ ^)^ whereas another identified a population of injury responsive *mpeg* ^*+*^ cells as lymphoid cells.^(^ ^62^ ^)^ Interestingly, we observed the presence of the TRAP^+^ punctae at the fracture site, which coincided with the recruitment of *mpeg1* ^+^ macrophages. These data suggest that *mpeg1* ^+^ cells recruited to fractures may differentiate into osteoclasts, or that mpeg1 may label a subpopulation of osteomac‐like cells.^(^ ^63^ ^)^ Monocytes are known to differentiate into osteoclasts under proinflammatory conditions in mammals, whereas WNT16 has been shown to inhibit the differentiation of bone marrow cells into osteoclasts *in vitro*.^(^ ^19^, ^48^ ^)^ In medaka, Rankl induction initiates the recruitment of *mpeg* ^*+*^ cells to bone before differentiating into osteoclasts.^(^ ^64^ ^)^ Additionally, the number of TRAP^+^ punctae at the fracture site 24 hpi and 4 dpi was significantly higher in *wnt16* mutants compared with WT. Taken together, these data pose the possibility that *mpeg1* is expressed by other HSC‐derived lineages such as osteoclasts, the differentiation of which may be regulated by Wnt16. However, whether distinct subpopulations of macrophages contribute differentially throughout fracture repair, and whether *mpeg1* is expressed by osteoclasts in zebrafish require further investigation.

In conclusion, our study helps to establish zebrafish as a strong, emerging model for studying factors influencing the dynamic behavior of the multiple cell types underpinning fracture repair and bone pathologies *in vivo*. By studying the lepidotrichia in the transparent fins of live zebrafish, we were able to visualize bone fragility phenotypes in a novel *wnt16* ^*−/−*^ mutant, as well as the influence of *wnt16* on bone repair in a dynamic, longitudinal manner. Using this model, we found evidence to suggest that the osteoporosis–associated gene *wnt16* elicits a protective effect against fracture susceptibility and promotes bone repair, potentially by buffering levels of canonical Wnt activity and promoting optimal osteoblast differentiation via *runx2a* and *osx*.

## AUTHOR CONTRIBUTIONS

**Lucy McGowan:** Conceptualization; data curation; formal analysis; investigation; methodology; resources; validation; visualization; writing‐original draft; writing‐review and editing. **Erika Kague:** Conceptualization; data curation; methodology; resources; visualization; writing‐review and editing. **Alistair Vorster:** Formal analysis; investigation. **Elis Newham:** Formal analysis; methodology; software; writing‐review and editing. **Stephen Cross:** Formal analysis; methodology; software; writing‐review and editing. **Chrissy Hammond:** Conceptualization; data curation; funding acquisition; project administration; resources; supervision; writing‐review and editing.

## Conflict of Interest

The authors declare no conflicts of interest.

### Peer Review

The peer review history for this article is available at https://publons.com/publon/10.1002/jbm4.10461.

## Supporting information


**Appendix S1.** Supplementary Information

## Data Availability

Mutant *wnt16* ^*−/−*^ zebrafish available upon request to Chrissy Hammond. Imaging data are available through the University of Bristol's RDSF server. Further information and requests for materials associated with this study should be directed to and will be made available upon reasonable request by the lead contact, Chrissy Hammond at chrissy.hammond@bristol.ac.uk.
