# Supplementary figures and images for: Wnt16 Elicits a Protective Effect Against Fractures and Supports Bone Repair in Zebrafish
Source: JBMR Plus. 2021 Feb 2;5(3):e10461. doi: 10.1002/jbm4.10461 (PMC7990157; doi:10.1002/jbm4.10461)

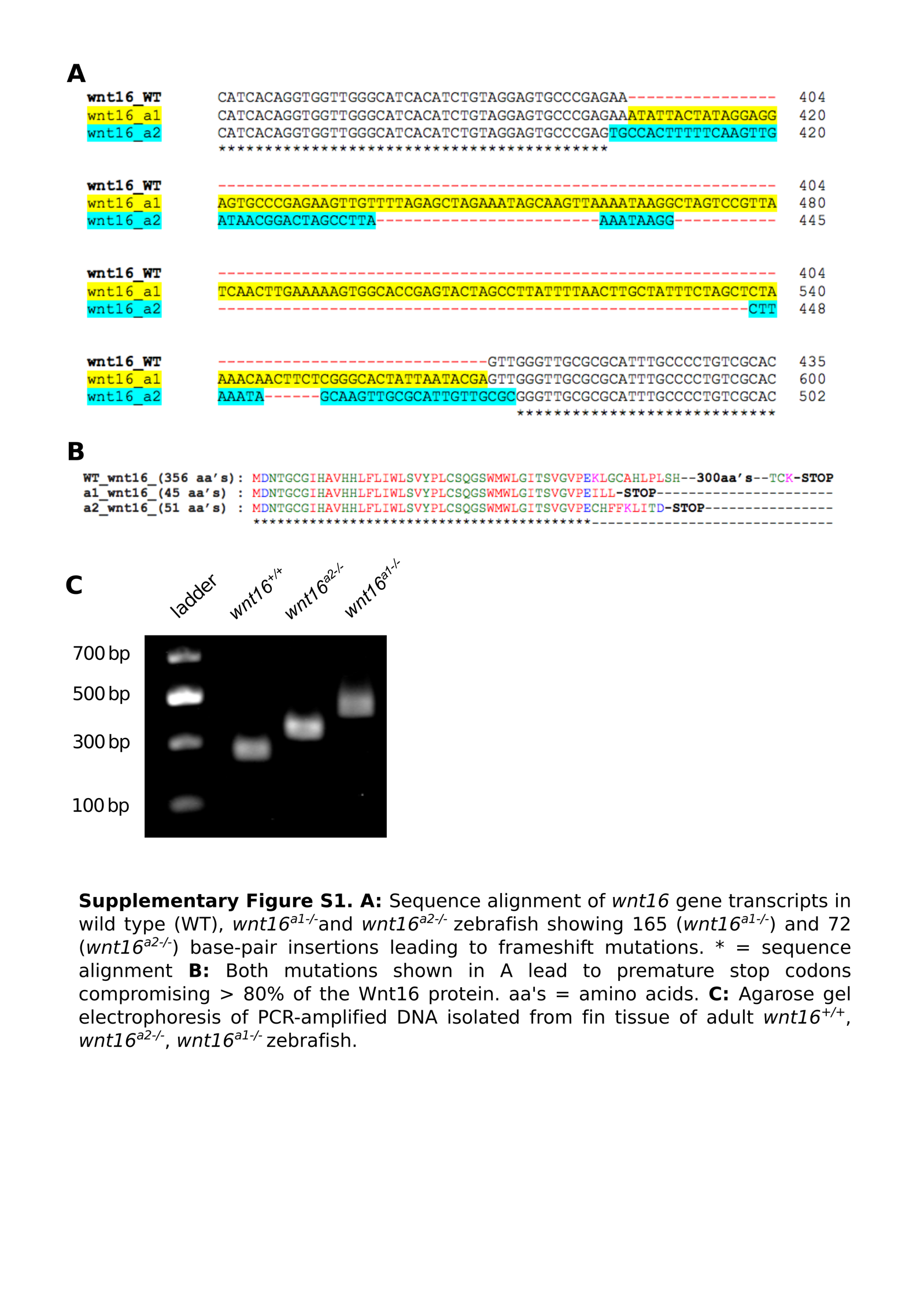


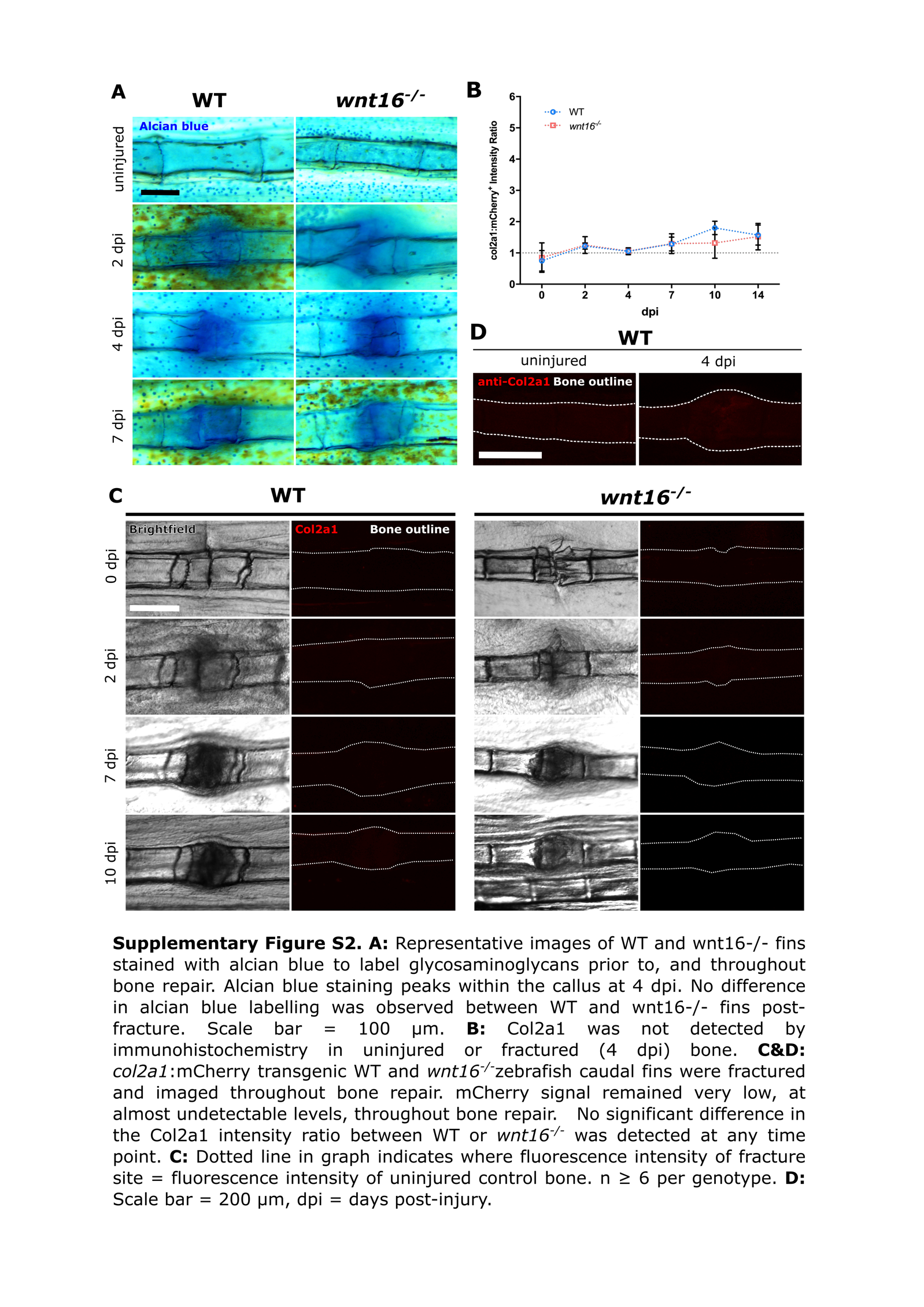


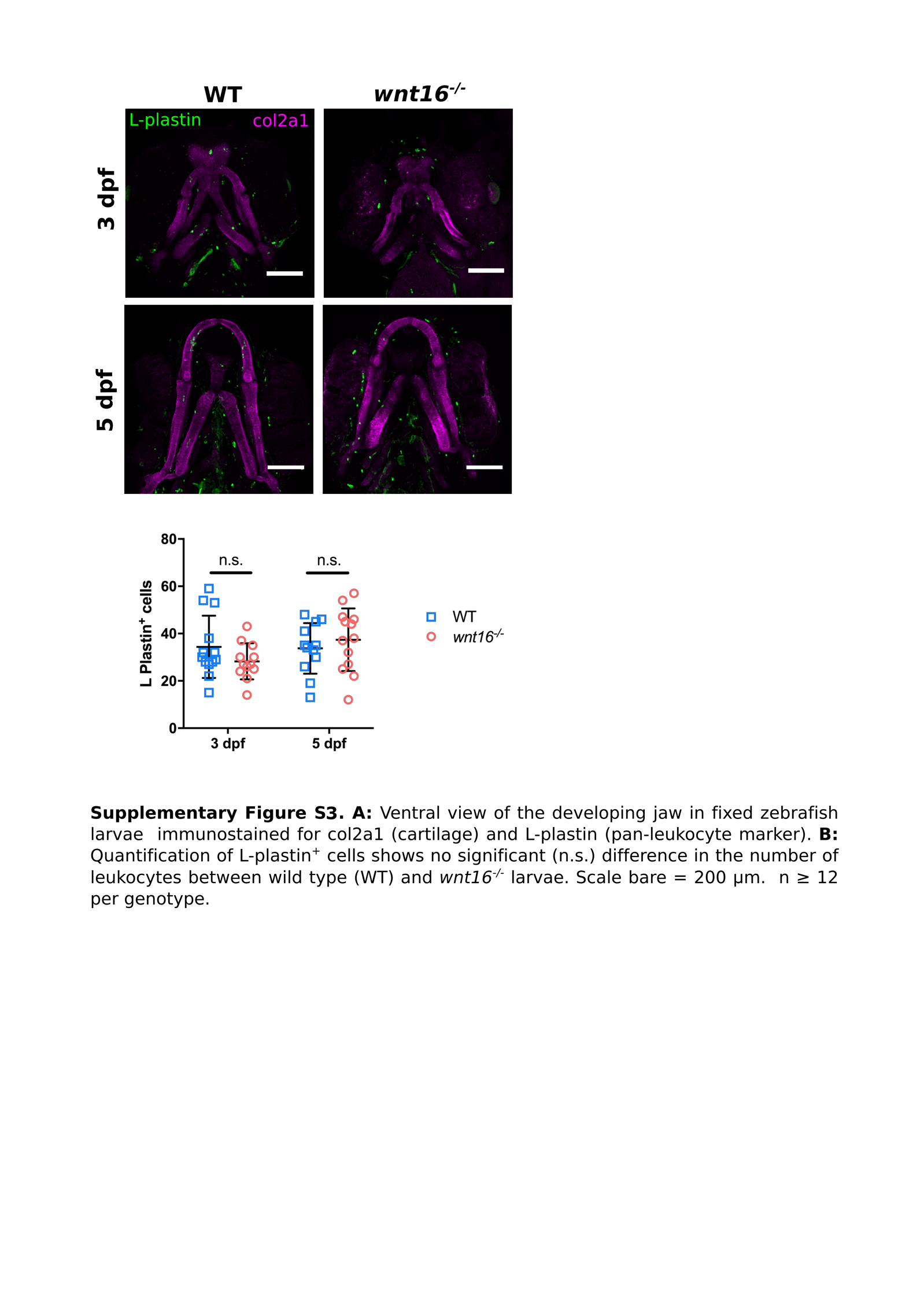


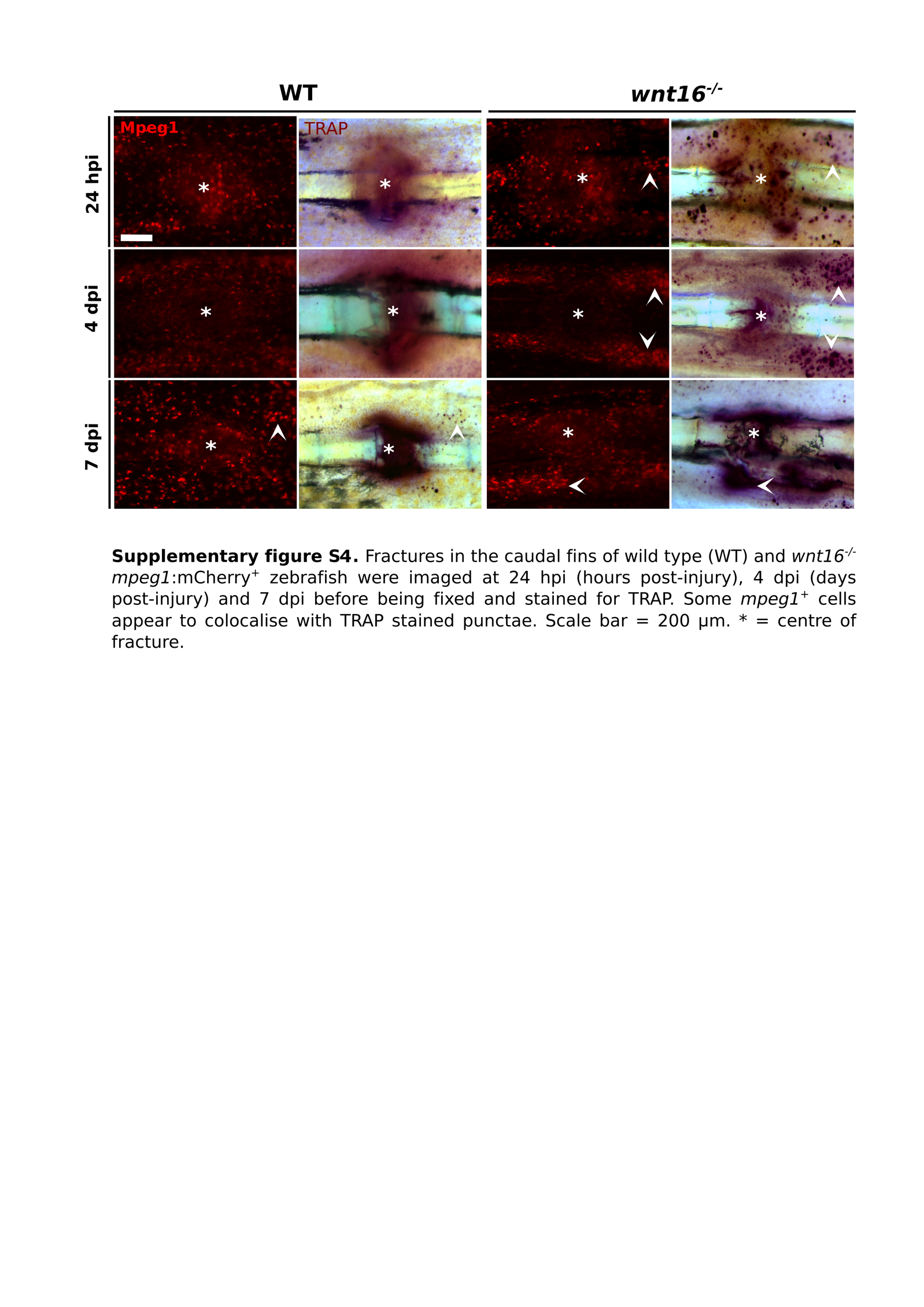

Supplement: Supplementary file 1 — Appendix S1. Supplementary Information [file JBM4-5-e10461-s001.docx]
